# The Evaluation of Sagittal Pelvic‐Femoral Kinematics in Patients with Cam‐Type Femoracetabular Impingement

**DOI:** 10.1111/os.13038

**Published:** 2021-08-19

**Authors:** Qing‐feng Yin, Jing Zhang, Tao Liang, Yu‐jie Liu, Shan‐xing Zhang, Chun‐bao Li

**Affiliations:** ^1^ Department of Orthopedics The Second Hospital of Shandong University Jinan China; ^2^ Department of Radiology, The Fourth Medical Center Chinese PLA General Hospital Beijing China; ^3^ Department of Orthopedics The First People's Hospital of Ningyang county Taian China; ^4^ Department of Orthopedics The Fourth Medical Center, Chinese PLA General Hospital Beijing China; ^5^ Department of Orthopedic Surgery The First Affiliated Hospital of Zhejiang Chinese Medical University Hangzhou Zhejiang Province China

**Keywords:** Femoroacetabular impingement, Sagittal pelvic‐femoral kinematics, True hip flexion

## Abstract

**Objective:**

To investigate the sagittal hip‐pelvic kinematics in symptomatic cam‐type femoroacetabular impingement (FAI) patients in the process of sitting down and compare their difference between patients with sitting pain complaint and those without.

**Methods:**

Twenty‐nine symptomatic cam‐type FAI patients were recruited from our clinic between May 2018 and October 2018. Patients were categorized into two groups depending on whether they complain of pain in prolonged sitting or not. The pelvic‐femoral measurements were assessed with a set of lateral pelvic radiography in sitting and standing respectively. Pelvic incidence (PI), sacral slope (SS), and proximal femoral shaft angle (PFSA) were measured on lateral pelvic radiography, and then pelvic tilting, apparent hip flexion, true hip flexion, and the pelvic‐femoral ratio were calculated to investigate the kinematic change from standing to sitting position. Demographic measurements, hip morphology measurements, functional measurements, visual analog scale (VAS), and pelvic‐femoral measurements were compared between the two groups.

**Results:**

Thirteen cases without sitting pain complaint and 16 cases with sitting pain complaint were stratified to Group N and Group P respectively. No was significant difference in age, body mass index (BMI), and gender between the two groups. Hip morphology measurements (α angle and lateral center‐edge angle) and functional measurements (iHOT‐12) showed no significant difference between the two groups. However, the mean VAS of pain while sitting was 0.5 ± 0.4 and 1.6 ± 0.6 in Group N and Group P respectively (*P* = 0.005). Patients with sitting pain complaint have increased pelvic PI compared to those without (50.1° ± 6.5° and 44.2° ± 7.6°, *P* = 0. 042). The changes in SS (pelvic tilting) from standing to sitting in Group N was significantly larger than that in Group P (21.8° ± 7.0° and 15.1° ± 6.5°, *P* = 0.012). Although no significant difference in apparent hip flexion and true hip flexion was found. Patients without sitting pain complaint demonstrated a higher pelvic‐femoral ratio (22.8% ± 7.9% and 16.1% ± 7.5%, *P* = 0.010) compared to those with sitting pain complaint.

**Conclusion:**

Sagittal pelvic‐femoral kinematics could have an influence on the symptomology of cam‐type FAI. The small PI and insufficient sagittal pelvic tilting in the process of sitting down could be related to the complaint of sitting pain in patients with symptomatic cam‐type FAI.

## Graphical abstract

What is the true hip flexion during the process of sitting down? The pelvis has a synergetic tilt backward while hip flexing, which could decrease the arc of hip flexion. Sagittal pelvic‐femoral kinematics could have an influence on the symptomology of cam‐type FAI. The small PI and insufficient sagittal pelvic tilting in the process of sitting down could be related to the complaint of sitting pain in patients with symptomatic cam‐type FAI.

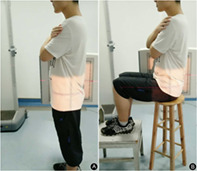

## Introduction

Femoroacetabular impingement (FAI) which has been recognized as the main cause of labrochondral disease and hip pain in young and middle‐aged adult populations could result in osteoarthritis and dysfunction of the hip, and cam deformity of the femoral head and pincer deformity of the acetabulum was considered as the critical pathology of FAI^1^, ^2^, ^3^, ^4^, ^5^. However, there is a large discrepancy between the prevalence of radiologic deformities and symptomatic FAI^6^, ^7^. For instance, some FAI patients with severe abnormal morphology but have mild even no clinical symptoms and *vice versa*. What makes this symptomatic discrepancy of FAI patient was not clear. The complaint of pain with prolonged sitting especially in a low‐seated position is common in symptomatic FAI. Low‐seated sitting was considered as the risky activity that could the symptom of FAI because high degree hip flexion could aggravate hip impingement. Besides morphologic abnormalities, the kinematic features of the hip joint could contribute to the presence of symptomatic FAI^8^. The flexion degree of femoracetabular joint (true hip joint) could be related to the symptom of FAI, but it has not been well studied.

Recently, the sagittal kinematics and rhythm of the spine‐pelvis‐femoral complex have become a new interesting field of study on FAI^9^, ^10^, ^11^, ^12^, ^13^, ^14^. Studies on hip‐pelvic kinematics revealed that patients with cam‐type FAI demonstrate less capability of pelvic tilt posteriorly during squatting comparing to healthy control^9^, ^15^, ^16^. Pelvic incidence (PI), a pelvic morphological parameter, was found to be related to the pelvic kinematics and a symptom of FAI, and a smaller PI was considered as a predictor for symptomatic FAI^10^, ^11^, ^14^. Fader *et al*. ^17^revealed the importance of lumbar lordosis in the progression of FAI symptoms and determined the difference in pelvic kinematics between symptomatic FAI and asymptomatic FAI. In brief, sagittal pelvic tilting has a synergistic effect related to hip flexion and could influence the true flexion arc of the hip in daily activity as sitting or squatting down. However, to what extent the sagittal pelvic tilting could affect the hip flexion in symptomatic FAI patients is not clear. We know little about the sagittal kinematics of the hip‐pelvic and the true flexion arc of the hip joint in the process of sitting down. The practical significance of research on sagittal pelvic kinematics was that we could distinguish symptomatic FAI patients with different hip‐pelvic kinematic patterns. The well‐directed protocol of conservative or surgical treatment for symptomatic FAI patients could be more efficient.

Therefore, we assessed the lateral pelvic radiographic from symptomatic FAI patients in our institute. The purpose of this study was: (i) to determine the sagittal hip‐pelvic radiographic measurements in standing and sitting position; (ii) to investigate the change of sagittal hip‐pelvic measurements in symptomatic FAI patients in the process of sitting down; and (iii) to compare the difference in sagittal hip‐pelvic kinematics between patients with sitting pain complaints and those without.

## Materials and Methods

### Study Design and Participants

This study was approved by the Institutional Review Board (No. 2018LW028). It was designed to recruit symptomatic FAI patients from our clinic between May 2018 and October 2018. All patients had informed consent.

### Inclusion Criteria

The inclusion criteria were: (i) patients aged between 18 and 50; (ii) patients diagnosed with symptomatic FAI; and (iii) patients with symptom duration exceed 6 months.

### Exclusion Criteria

Patients were excluded if they presented: (i) history of hip or spine surgery; (ii) lumbar or sacral deformity; (iii) ankylosing spondylitis; (iv) pincer deformity; (v) dysplasia; (vi) Tönnis grade ≥ 2; and (vii) other diseases that could result in hip pain.

Fifty‐two consecutive subjects met inclusive criteria were screened, two subjects were excluded for the history of hip surgery, one subject was excluded for the coexistence of osteoid osteoma, one subject was excluded for ankylosing spondylitis, two subjects were excluded for borderline dysplasia, and 17 patients with pincer deformity of FAI were also excluded. Finally, 29 symptomatic cam‐type FAI patients were enrolled.

Each participant was asked, “Have you been disturbed by hip pain or discomfort with prolonged sitting (>30 min) in daily life?” Patients without prolonged sitting pain and patients with prolonged sitting pain were stratified to Group N and Group P respectively.

### Clinical Evaluation

All participants were assessed comprehensively by a senior surgeon. The diagnosis of FAI was made according to the triad of symptoms, physical examination, and imaging findings.

#### Visual Analog Scale (VAS)

The visual analog scale is a simple measurement for pain intensity using a continuous scale with a length of 10 cm. The left end of the scale labeled 0 indicates no pain, and the right end labeled 10 indicates most severe pain. Each participant was asked to take a prolonged sitting (>30 min) and mark on the scale to reflect the pain severity they experienced. Pain VAS is a subjective indicator reflecting the level of the patient's complaints.

#### International Hip Outcome Tools‐12 (iHOT‐12)

International hip outcome tools‐12 is a patient‐report outcome questionnaire developed by Multicenter Arthroscopy of the Hip Outcomes Research Network^18^, which includes 12 questions about the patient's symptoms, functional limitations, and social, emotional and occupational limitations. Each question was followed by a VAS ranged from 0 to 100, the patient was asked to answer each question by marking on the scale to reflect their limitation in this term. The iHOT‐12 score is calculated by averaging all scores. iHOT‐12 provides an overall assessment of the patient's hip function.

### Radiography and Measurement

**Fig 1:**
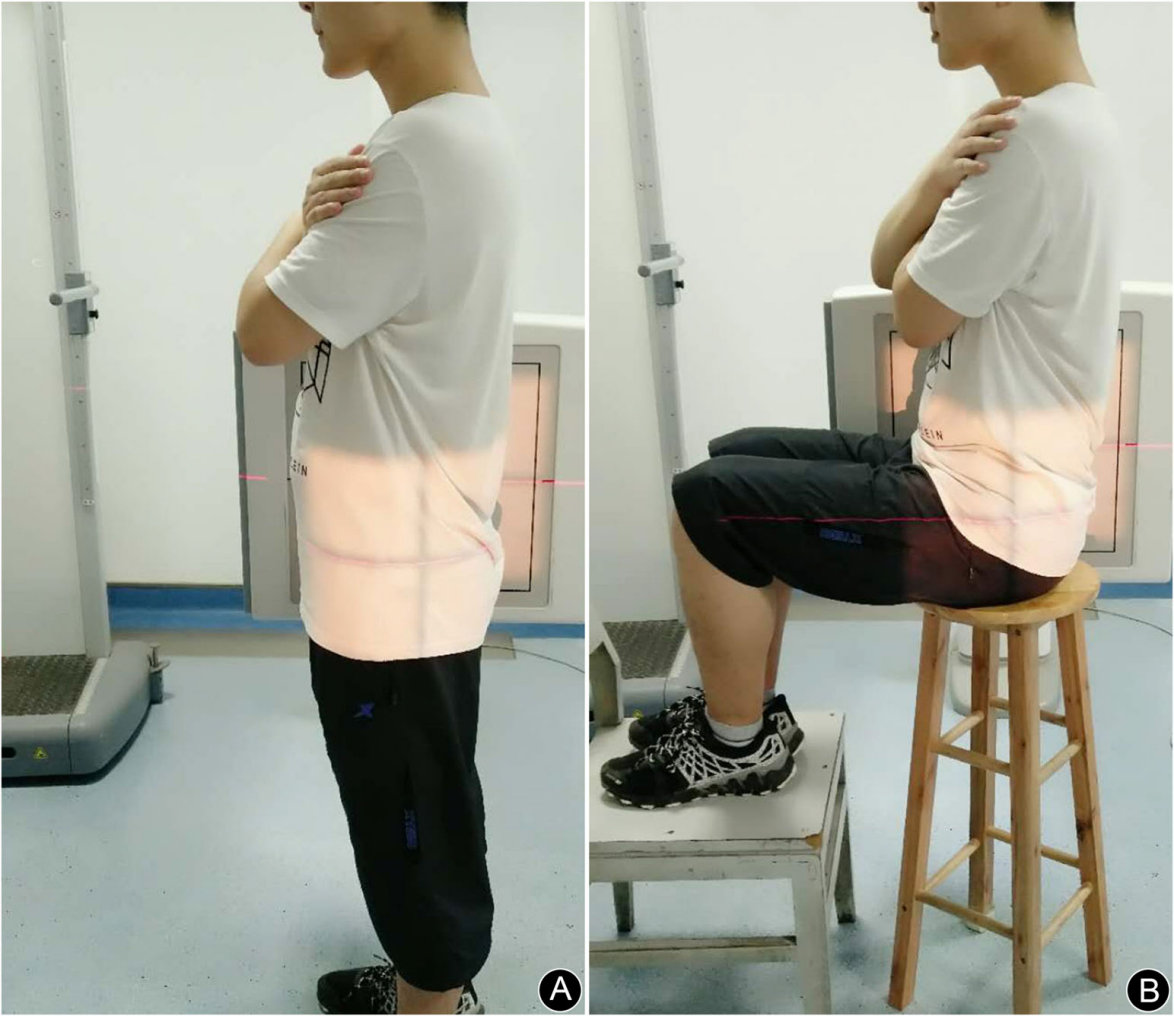
Lateral views of pelvic‐femoral radiography was taken in the standing position (A) and in the sitting position (B) respectively.

Each participant has taken the standard anterior–posterior (AP) pelvic radiography and the Dunn view of the hip. Additional lateral pelvic radiographs both in the sitting and standing position were arranged for each participant to detect the femoral‐pelvic kinematics (Fig. 1). The sitting and standing position was standardized with their hands resting on their chest, which has been described as a reproducible and reliable method^19^, ^20^. All plain radiographs were taken with a Discovery XR656 Plus instrument (GE Healthcare, Little Chalfont, UK) by a well‐trained senior radiologist, that guaranteed standard radiography could be obtained. All images were saved and measured in the picture archiving and communication system. All radiographs were independently evaluated in a standardized method by two fellowship‐trained surgeons. If excellent reliability was not achieved, a senior examiner made the final decision. The measurements were prescribed as follows.

#### Lateral Center‐Edge Angle (LCEA)

The lateral center‐edge angle, which is formed by a vertical line perpendicular to the transversal line of the pelvis and a line connecting the center of the femoral head through the most lateral portion of the acetabulum, was measured on the AP pelvic radiograph in the standing position. Normal acetabular coverage was defined with LCEA between 25° and 38°, The pincer lesion was defined with LCEA ≥ 39°. Hip dysplasia was defined with LCEA < 20°.

#### α Angle

The α angle was measured on the Dunn view radiography, which is the angle formed by the central axis of the femoral neck and the radius line where the femoral head loses its sphericity. The radiologic cam deformity was defined as α angle α ≥ 55°.

#### Pelvic Incidence (PI)

The pelvic incidence (PI) was measured between the midpoint of the line perpendicular to the sacral plate and the line connecting this point to the middle of the bicoxo‐femoral axis^21^. PI is a morphological measurement, which indicates the feature of pelvis and dose not vary with posture.

#### Sacral Slope (SS)

The sacral slope (SS) was measured *via* the angle subtended by a horizontal reference line and a tangent lined to the sacral endplate^22^. The change of SS indicates the pelvic tilting in the sagittal plane during the process of sitting down.

#### Proximal Femoral Shaft Angle (PFSA)

The proximal femoral shaft angle (PFSA) was defined as the angle subtended by the proximal axis line of the femoral shaft and the vertical line. PFSA is a postural measurement that indicates the posture of the femoral shaft in the sagittal plane.

#### Apparent Flexion of the Hip

Apparent flexion of the hip joint which has the definition of the visual appearance of hip flexion was calculated as the change in PFSA between standing and sitting. It indicates the flexion of the femoral shaft in the sagittal plane during the process of sitting down.

#### True Hip Flexion

True flexion of the hip defined as the flexion degrees of femoracetabular joint was calculated by apparent flexion subtracting pelvic tilting. It could be influenced by the flexion of the femoral shaft and the tilt of the pelvis.

#### Pelvic‐Femoral Ratio

Pelvic‐femoral ratio was calculated as the percentage of pelvic tilting contributing to the whole apparent hip flexion. It indicates the pelvic‐femoral flexibility and compensatory capacity of the pelvis.

**Fig 2:**
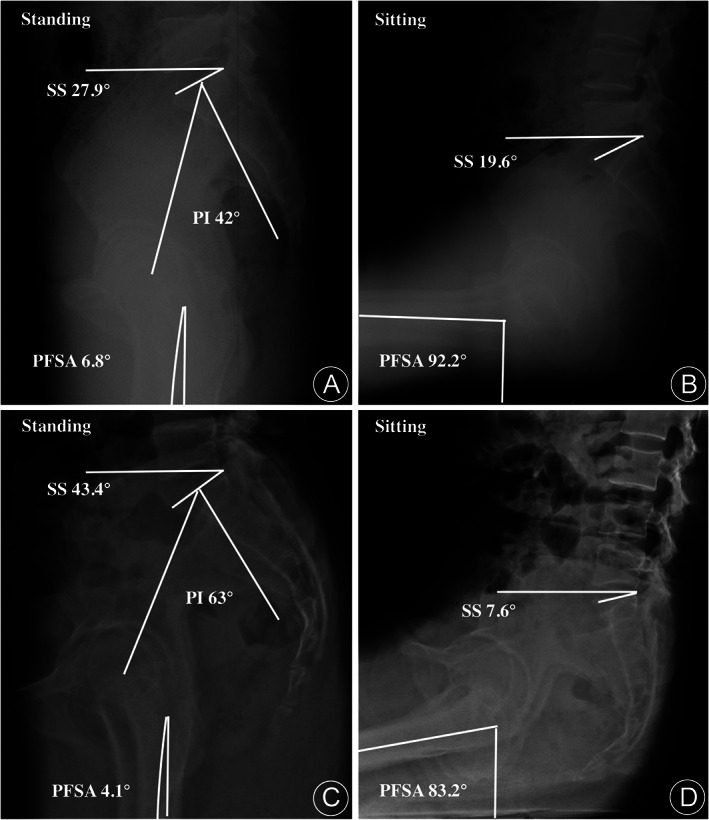
Sacral slope (SS), proximal femoral shaft angle (PFSA), and pelvic incidence (PI) were measured on lateral views of pelvic‐femoral radiography. A 54‐year‐old male patient with sitting pain complaint was measured in the standing position (A) and in the sitting position (B). PI = 42°, pelvic tilting 8.3° = 27.9°–19.6°, apparent hip flexion 85.4° = 92.2°–6.8°, true hip flexion 77.1° = 85.4°–8.3°. A 36‐year‐old female patient without sitting pain complaint in the standing position (C) and in the sitting position (D) (PI = 63°, pelvic tilting 35.8° = 43.4°–7.6°, apparent hip flexion 79.4° = 83.2°–4.1°, true hip flexion 43.6° = 79.4°–35.8°).

For example, a case showed the change in proximal femur shaft angle (apparent hip flexion) is 85.4° = 92.2°–6.8°, and the change in sacral slope (pelvic tilting) is 8.3° = 27.9°–19.6°, true hip flexion arc is 77.1° = 85.4°–8.3°. The pelvic‐femoral ratio is 11.2% = 8.3°/85.4°. (Fig. 2).

### Statistical Analysis

All statistical analyses were conducted using R software from CRAN (R Foundation for Statistical Computing, Welthandelsplatz, Vienna, Austria). All data were expressed as the mean standard ± deviation. Demographic measurements including age, BMI, α angle, LCEA, VAS, and iHOT‐12 and all pelvic‐femoral angular parameters including PI, SS, and PFSA following the normal distribution were compared using the Student *t*‐test, and as a categorical variable, the gender was expressed by number and compared using the chi‐square test, *P* < 0.05 was considered statistically significant.

## Results

### Demographic Results

Twenty‐nine symptomatic cam‐type FAI patients (17 males and 12 females) were enrolled in this study with a mean age of 36.4 years (range, 27–50 years) and a mean body mass index (BMI) of 25.2 (range, 19.3–37.2) kg/m^2^. Thirteen cases without complaint of prolonged sitting pain and 16 cases with a complaint of prolonged sitting pain were stratified to Group N and Group P respectively. There is no significant difference in age, BMI, and gender. Group N and Group P. (*P* = 0.581, 0.573, and 0.773). The mean α angle in Group N and Group P was 59.8° ± 4.5°and 61.8° ± 5.5° respectively (*P* = 0.289). The mean LCEA was 31.4° ± 3.6° and 30.8° ± 2.8° in Group N and Group P respectively (*P* = 0. 677) (Table. 1).

**TABLE 1:** Demographical comparison of cam‐type FAI patients

Characters	Group N (N = 13)	Group P (N = 16)	*P*‐value
Gender (M/F)	8/5	9/7	0.773
Age (years)	35.5 ± 7.6	37.3 ± 9.6	0.581
Body mass index (kg/m^2^)	24.8 ± 3.7	25.6 ± 4.2	0.573
α angle (°)	59.8 ± 4.5	61.8 ± 5.5	0.289
Lateral center‐edge angle (°)	31.4 ± 3.6	30.8 ± 2.8	0.677
Visual analogue scale	0.5 ± 0.4	1.6 ± 0.6	0.005
iHOT‐12 score	43.6 ± 9.7	40.6 ± 9.4	0.354

Data are expressed as mean ± SD and number.

iHOT‐12, international hip outcome tool‐12.

### Functional Outcomes

#### iHOT‐12

No significant difference in the iHOT‐12 score between the two groups was evident. The score was 43.6 ± 9.7 and 40.6 ± 9.4 in Group N and Group P respectively (*P* = 0.354).

#### Visual Analog Scale (VAS)

The average VAS of pain while sitting was 0.5 ± 0.4 and 1.6 ± 0.6 in Group N and Group P respectively. Pain VAS reported by patients with sitting pain complaints was 2.2 times higher compared to that in patients without sitting pain complaints. The difference between the two groups was statistically significant (*P* = 0.005).

### Pelvic‐Femoral Radiological Results

#### Pelvic Incidence (PI)

A significantly increased mean of PI could be found in Group N comparing to that in Group P (50.1° ± 6.5° and 44.2° ± 7.6°, *P* = 0. 042). The mean of PI in Group N was 13.34% larger than that in Group P.

#### Sacral Slope (SS)

Whether in a sitting or standing position, the comparison of SS did not show a significant difference between the two groups. However, the pelvic tilting backward from standing to sitting in Group N was significantly larger than that in Group P (21.8° ± 7.0° and 15.1° ± 6.5°, *P* = 0.012). The pelvic tilting in Group N was 44.37% higher than that in Group P.

#### Proximal Femoral Shaft Angle (PFSA)

The mean PFSA (standing) in Group N and Group P was 5.6° ± 2.1° and 4.3° ± 2.1°respectively while standing (*P* = 0.250). The mean PFSA (sitting) in Group N and Group P was 99.8° ± 9.1° and 94.1° ± 11.0° respectively while standing (*P* = 0.139).

#### Apparent Hip Flexion

There was no significant difference in apparent hip flexion between the two groups. The mean change of *PFSA* (apparent hip flexion arc) in Group N and Group P was 94.2° ± 8.8° and 89.8° ± 11.8° respectively (*P* = 0.281).

#### True Hip Flexion

The mean of true hip flexion in Group N and Group P was 72.4° ± 11.7° and 74.7° ± 16.2° respectively (*P* = 0.671).

#### Pelvic‐Femoral Ratio

Patients in Group N demonstrated a higher pelvic‐femoral ratio (22.8% ± 7.9% and 16.1% ± 7.5%, *P* = 0.010) compared with those in Group P during the process of sitting down. The compensatory capacity of pelvis in Group N was 41.61% higher than that in Group P (Table. 2).

**TABLE 2:** The pelvic‐femoral measurements of cam‐type FAI patients

Parameters	Group N (N = 13)	Group P (N = 16)	*P*‐value
Pelvic incidence (°)	50.1 ± 6.5	44.2 ± 7.6	0.042
Sacral slope (standing) (°)	40.4 ± 7.4	37.6 ± 7.5	0.451
Sacral slope (sitting) (°)	18.6 ± 7.5	22.5 ± 4.5	0.138
Pelvic tilting (change in sacral slope) (°)	21.8 ± 7.0	15.1 ± 6.5	0.012
PFSA (standing) (°)	5.6 ± 2.1	4.3 ± 2.1	0.250
PFSA (sitting) (°)	99.8 ± 9.1	94.1 ± 11.0	0.139
Apparent hip flexion (change in PFSA) (°)	94.2 ± 8.75	89.8 ± 11.8	0.281
True hip flexion (°)	72.4 ± 11.7	74.7 ± 16.2	0.671
Pelvic‐femoral ratio (%)	22.8 ± 7.9	16.1 ± 7.5	0.010

Data are expressed as mean ± SD.

PFSA, proximal femoral shaft angle.

## Discussion

The present study was designed to investigate hip‐pelvic kinematic features in symptomatic cam‐type FAI patients. The results show that patients who complain of sitting pain have a decreased PI and demonstrate less capability of pelvic tilting backward during sitting down compared to these without sitting pain complaint. Moreover, patients with sitting pain complaints were found to be stiffer in pelvic‐femoral rhythm.

### Pelvic Tilting and Sagittal Kinematics

Most of the time, the pelvis has a synergetic tilt backward while hip flexing, which could prevent the contact cam lesion of the femoral head and acetabular rim. We found the apparent hip flexion was 94.2° on average, in patients without sitting pain complaint while the true hip flexion was 72.4°. What we found was that pelvic tilting contributes 21.8° on average in the process of sitting down to alleviate the collision of the femoral head and acetabulum. However, the contribution of pelvic tilting in patients with sitting pain complaint was only 15.1° on average. Our results have reasonable concordance with the findings of Fader *et al* .^17^, and they found the change in sacral slope from standing to sitting was 12° and 18° in symptomatic FAI and asymptomatic FAI respectively. Insufficient compensatory pelvic tilting in the process of sitting down could result in truer hip flexion and more severe symptoms for FAI patients.

Several studies have reported the pelvic tilting during hip flexion, but whether it is causative or effective has not been clearly explained. Van Houcke *et al*.^23^ found the posteriorly pelvic tilting in FAI patients was significantly increased compared with healthy control during active hip flexion, and pelvic tilting was considered as an active compensatory effect. While Fernquest *et al*.^24^ found the degree of pelvic tilt during passive hip flexion was related to the size and location of cam lesion, that could indicate the pelvic tilting is secondary to the hip impingement. Bagwell *et al*. ^9^ revealed that patients with cam lesion could not squat as low as healthy control. Excessive hip flexion was considered as a provocative factor for symptomatic FAI. In the present study, we did not find patients who complained of sitting pain have more compensatory pelvic tilting. Although we could not determine the pelvic tilting is causative, it seems that the insufficient pelvic tilting backward could be an aggravating factor for pain in symptomatic cam‐type FAI patients.

### Pelvic Incidence and Sagittal Kinematics

As a morphologic parameter, pelvic incidence (PI) was considered to be a critical factor that could influence pelvic tilt and was related to the symptom of FAI. Previous anatomical and radiological studies have found that patients with FAI deformity have smaller PI compared with healthy control^14^, ^15^, ^16^. Fader *et al*.^17^ compared the PI value in symptomatic FAI and asymptomatic FAI and found they are 53°and 48° on average respectively. Our study investigated the difference in PI between different symptomatic cam‐type FAI. We found the overall mean PI of this cohort was 46.8°, and that in symptomatic cam‐type FAI patients with and not complaining of sitting pain was 44.2° and 50.1° respectively. The mean PI in our cohort from a Chinese population has good consistency to that in Fader's^17^ cohort from a western population (46.8° *vs* 48°).

### Pelvic Kinematics and Daily Activities

Being different from previous studies investigating pelvic kinematics during squatting down using an electromagnetic tracking device ^9^, ^12^, ^15^, ^16^. Our research using the lateral radiography investigated the kinematics of the pelvis from standing to sitting. That is because pain with prolonged sitting is a common complaint of symptomatic FAI. Therefore, investigation on sagittal kinematics of the pelvis during sitting down could facilitate the enhancing of understanding of FAI etiology. Moreover, the evaluation of pelvic‐femoral measurement with a set of common lateral pelvic radiography could be more feasible in clinical practice, and it could supply additional kinematic information for surgeons to make a comprehensive treatment for symptomatic cam FAI.

The current study has some limitations. First, the present study only involved symptomatic cam‐type, which may limit the generalization of the findings, but it followed the previous studies reported by Fernquest *et al*.^24^ and Bagwell *et al*.^9^. Cam‐type was a classical subtype of FAI with well‐understood pathomechanism, it is rational to select cam‐type as the representation of FAI subjects. Moreover, if all subtypes of FAI with different characteristics and mechanisms were included, more confounding factors could be added to this study. Second, the absence of a healthy control group could be one weakness of this study, but it does not impair the reliability of the study. That is because the aim of the study was to detect the difference in pelvic‐femoral kinematics in symptomatic FAI, and we should eliminate the influence of hip morphology, therefore, symptomatic FAI without pain in prolonged sitting was defined as the control group. Third, the sample size of the current study is limited, but not less than previously published studies^17^.

### Conclusion

Sagittal pelvic‐femoral kinematics could have an influence on the symptomology of cam‐type FAI. The small PI and insufficient sagittal pelvic tilting in the process of sitting down could be related to the complaint of sitting pain in patients with symptomatic cam‐type FAI. The clinical relevance of study on sagittal pelvic‐femoral kinematics should be investigated in future.
